# Effects of ethanol on non‐invasively recorded cerebellar, cerebral, and postural responses to axial perturbation: A case study

**DOI:** 10.14814/phy2.70897

**Published:** 2026-04-29

**Authors:** Neil P. M. Todd, Sendhil Govender, James G. Colebatch

**Affiliations:** ^1^ UNSW Clinical School Randwick Campus Sydney New South Wales Australia; ^2^ Neuroscience Research Australia UNSW Sydney New South Wales Australia

**Keywords:** cerebellum, electro‐cerebellogram (ECeG), ethanol, postural reflex

## Abstract

We report for the first time non‐invasively recorded ethanol (EtOH) induced changes in the spontaneous and evoked activity of the human cerebellum. We recorded electroencephalography (EEG), electro‐cerebellography (ECeG), lower limb electro‐myography (EMG), and posturography in a 64 year old adult male before and after oral ingestion of EtOH while standing at rest and in response to axial perturbation of the trunk. The a xial perturbation is known to give rise to a well‐defined postural reflex of likely brain‐stem origin in lower‐limb muscles accompanied by correlated short and long latency cerebral and cerebellar responses. The associated cerebellar response is of likely climbing fiber (CF) origin, characterized by post‐CF inhibition of the spontaneous Purkinje cell (PC) activity, non‐invasively manifest as pausing in the high‐frequency ECeG. In our case, EtOH reversibly attenuated the postural reflex and associated cerebral responses, whilst also causing an increase in the spontaneous high‐frequency ECeG and severely disrupting the post‐CF pausing of the ECeG in response to axial perturbation. The initial component of the CF response was unaffected, however, likely reflecting the afferent volley to the perturbation. These effects demonstrate that non‐invasive recordings of cerebellar electrophysiology are possible and can provide important pathophysiological insights.

## 1 INTRODUCTION

From the earliest cerebellar research, electrical measurements of spontaneous activity in or on the surface of the cerebellum, referred to as the electrocerebellogram (ECeG), revealed its high‐frequency character when compared to EEG, typically in the range of about 150–250 Hz (Adrian, 1935; Dow, 1938). Recent developments in non‐invasive electrophysiology have shown that, contrary to prior belief (Andersen et al., 2020), activity arising from the posterior lobe of the cerebellum is detectable using EEG and MEG sensors on the scalp (Andersen et al., 2020; Todd et al., 2017). In our prior work we have shown that it is possible to record both cerebellar evoked potentials (CEPs) and the spontaneous activity of the cerebellum, the ECeG, non‐invasively (Govender et al., 2024; Todd et al., 2017, 2018a, 2018b, 2019). While some of these early results have been replicated, for example, (Latorre et al., 2025; Romero et al., 2025), the view remains that non‐invasive electrophysiology is non‐viable, either due to fine anatomical cerebellar folding and cancellation or occlusion by neck muscle EMG (Andersen et al., 2020). Direct recordings from the exposed human cerebellar cortex or deep nuclei would provide a theoretically ideal opportunity to study their electrophysiology (Dalal et al., 2013; Neidermeyer & Uematsu, 1974), but this is extremely difficult. Another opportunity to evaluate human cerebellar electrophysiology might also be available from study of patients with a malformed or absent cerebellum, such as cerebellar agenesis (Bounanani et al., 2024). These cases are, however, extremely rare and interpretation controversial due to associated complications (Bounanani et al., 2024).

Due to the above difficulties we sought an alternative approach to providing evidence to judge the validity of the non‐invasive ECeG by means of ethanol (EtOH) intoxication (Mitoma et al., 2021). EtOH is known to have a profound effect on the cerebellum, primarily, but not exclusively, via its positive allosteric modulation of γ‐aminobutyric acid A (GABA‐A) receptors, giving rise to many of the common symptoms of EtOH intoxication, including ataxia, dysmetria, dysarthria, and motor and cognitive dysfunction (Mitoma et al., 2021; Rossi, 2023). GABAergic inhibition plays a critical role in the control of cerebellar function, directly in Purkinje cell (PC) output, and via both molecular layer inter‐neuronal (stellate and basket cell) modulation of PCs and Golgi cell modulation of mossy fiber input (Mitoma et al., 2021; Rossi, 2023). Thus, the resultant effects are complex, depending on dose and whether acutely or chronically administered. In small doses acute EtOH administration can give rise to an increase in simple spike (SS) activity (Zhang et al., 2017), whereas chronic EtOH administration can impair SS activity (Dong et al., 2021). We should thus expect to observe changes in spontaneous ECeG with dose, which is a manifestation of SS activity. EtOH administration is also known to disrupt CF‐responses and post‐CF pausing of SS activity, which should also be observable on post‐CF pausing of ECeG (Zhang et al., 2017).

For the present study we chose to examine effects of EtOH on a well‐described postural response to axial taps (“mini‐perturbations”) and associated changes in cerebral and cerebellar electrophysiology (Marsden et al., 1970; Traub et al., 1980). The response comprises short latency excitatory and inhibitory responses in lower‐limb muscles accompanied by cerebellar and cerebral potentials and changes in the high frequency ECeG, the latter characteristic of CF responses and post‐CF pausing (Colebatch et al., 2016; Heine et al., 2020; Todd et al., 2021; Govender et al., 2024). We hypothesized that EtOH would cause a dose dependent but reversible disruption of cerebellar function with possible disruption of the evoked potentials, resting high frequency ECeG or CF modulations.

## 2 METHODS

This case study was prepared following the CARE guidelines (CAse REport guidelines); a completed CARE checklist is provided as a File S1.

### 2.1 Volunteer and procedure

**FIGURE 1:**
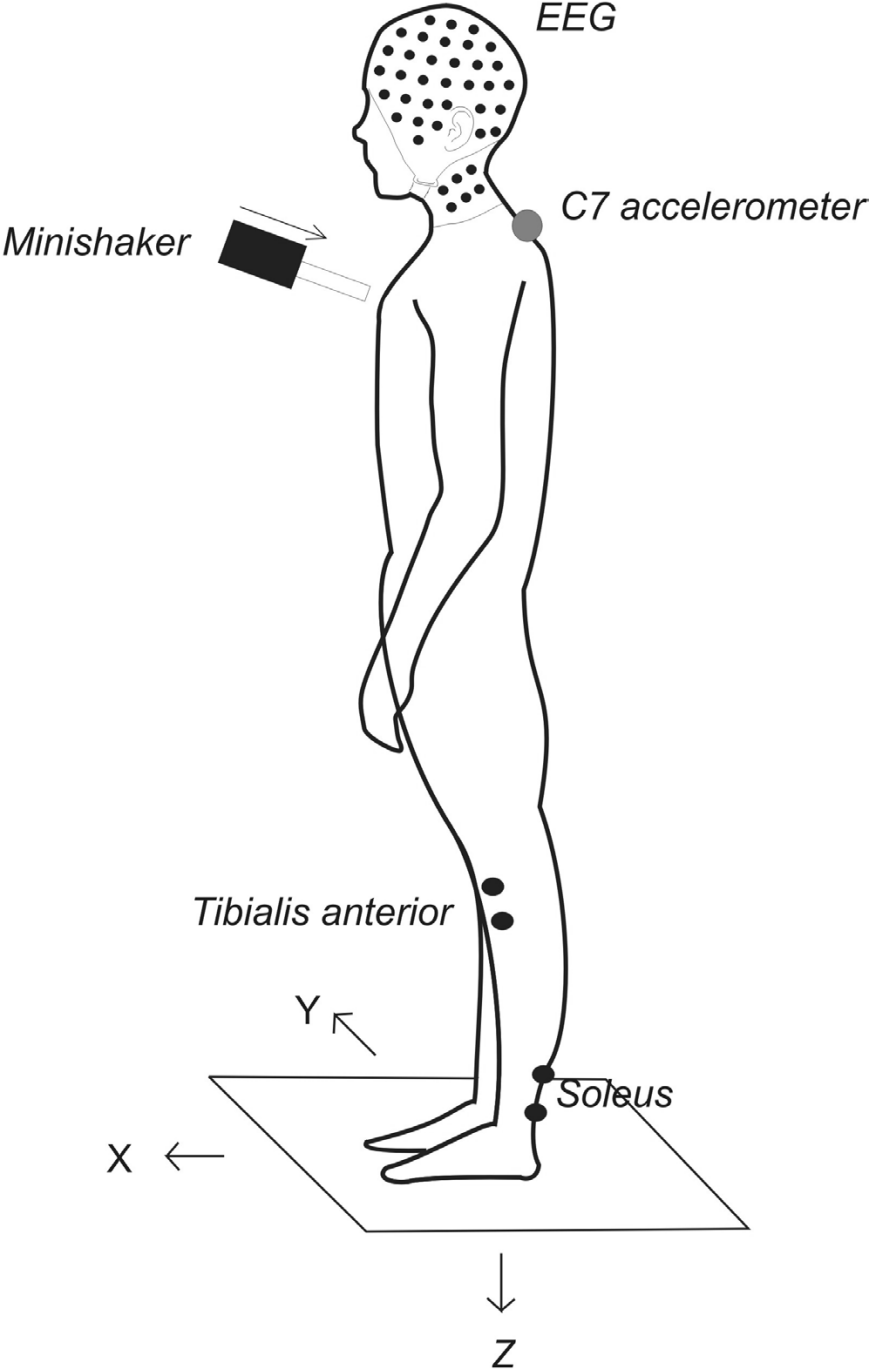
Experimental setup used during mini‐perturbation stimulation. Stimuli were delivered to each subject's upper sternum using a minishaker device. EMG was recorded from the tibialis anterior (TA) and soleus (SOL) muscles bilaterally at rest and with backwards lean during stimulation. Cephalic recordings were made of EEG activity using a customized cap which extended to Iz and below to the upper neck at SIz and Bz levels. Light gray circle indicates the linear accelerometer position at the trunk (C7). A force plate recorded force in 3‐D (X, Y, Z) at the four corners of the plate. The moment about the Y‐axis (My) was computed from a*(−fZ1 + fZ2 + fZ3‐fZ4), where *a* = sensor offset from the midpoint on the X‐axis (200 mm).

A 64 year old, 86 kg male volunteer (one of the three authors) with no prior neurological or balance dysfunction (and known over many years of electrophysiological recording to have robust and healthy neurophysiology) gave his informed verbal consent for the procedure, which received ethical approval from the South Eastern Sydney Local Health District Human Research Ethics Committee. The study was conducted in a single experimental session and consisted of five recording blocks, lasting about 3 ½ h, under medical supervision throughout. In each of the five blocks, electrophysiology and posturography were recorded, with the volunteer standing at rest on a force plate then subject to the axial perturbations applied to the sternum (Figure 1). The first block was recorded before EtOH, the second block 10 mins after administration of 20 g (55 mL of 46%), the third block 10 mins after a 2nd dose of 145 mL (total 200 mL 46% EtOH), the fourth after 1 h following 2nd dose and the fifth 2 ½ hours following the 2nd dose. The initial dose would give a blood alcohol concentration (BAC) of about 0.033% using the Widmark formula for this individual, just below the legal limit for driving. With no elimination, the total dose would give a BAC of about 0.12%, well over the limit. The Widmark estimates were supplemented by means of breath alcohol concentration (BrAC), estimated using a commercial breathalyzer (Sigma Healthcare) with regular measures taken over the whole session. After ingestion of the first dose, neurological assessments for cerebellar and vestibular symptoms were made between recordings. The volunteer was kept under supervision several hours after the 2nd dose and discharged after the BrAC had dropped below 0.05%.

### 2.2 (Mini)‐perturbations

The “mini‐perturbation” consisted of a brief, smoothed impulsive acceleration with an exponential decay. It consisted of a 3rd order gamma waveform with a 4 ms rise time and an exponential decay, with a full width at half maximum of 10.7 ms. These impulses were generated using a laboratory interface (CED Power1401, Cambridge Electronic Design, Cambridge, UK), a power amplifier (model 2718, Brűel & Kjær, Denmark), and customized software. The stimulus was delivered using a hand‐held mini‐shaker device (model 4810, Brüel & Kjaer P/L, Denmark) with an attached cylindrical perspex rod (diameter: 2.5 cm, length: 9.2 cm). The mini‐shaker was applied to the upper sternum with the initial displacement of the rod towards the subject (positive polarity) at a fixed intensity of 20 volts peak (~14 N peak force level (FL)).

### 2.3 Posturography, acceleration, and EMG

Responses to perturbations applied at a rate of 1–2 Hz were recorded with 100 individual trials for each condition. In each block, the volunteer was recorded standing at rest on the force plate (Kistler Instrument, Switzerland) with feet in a fixed position, determined by markers on the force plate, and then subject to the axial perturbations with feet in the same position but with a backwards lean, monitored by the experimenter for consistency. A uniaxial accelerometer was positioned at the vertebra prominens (C7). Unrectified EMG was recorded bilaterally from the TA and soleus muscles. Active electrodes were positioned 1–2 cm above the musculotendinous junction for soleus and 1–2 cm lateral to the tibia for TA. Reference electrodes were placed 2 cm below the active electrodes. Due to the posture adopted, tonic EMG activity in soleus was low and it was not analyzed in detail. A ground electrode was placed on the midpoint of the left lower leg. EMG signals were amplified (2500×) and filtered (8 Hz to 1.6 kHz) using AA6 Mk III amplifiers (Medelec Ltd., Old Woking, Surrey UK) and recorded using Signal software and a Power1401 (Cambridge Electronic Design, Cambridge, UK). EMG was rectified and averaged offline. All recordings were sampled at 4096 Hz from 0.2 s prior to the stimulus onset to 0.8 s after it.

### 2.4 EEG recordings

EEG was recorded in parallel using a 10–10 cerebellar‐extended cap (EASYCAP GmbH, Germany) which consisted of 96 EEG electrodes, formed by completing the inion (Iz) circumference of the standard 10–10 64 channel system and adding two 10% circumferences below Iz, labeled the sub‐inion (SIz) and base (Bz) circumferences, in order to provide full coverage over the posterior fossa (Heine et al., 2020). Recordings were made using an ActiveTwo AD‐box amplifier and ActiView software (BioSemi, Holland). In both rest and stimulus conditions, triggers were recorded at the onset of the stimuli. Ascii exports made around the trigger points using BESA software (version 7.1, MEGIS Software GmbH, Germany), referenced to a common average. These were read and converted to CFS format using Matlab (Mathworks Australia P/L) custom routines. Peaks are named following Govender et al. (2024).

### 2.5 Spectral power analysis

After recording EEG/ECeG and epoching, we performed spectral power analyses on midline channels over a 2.0 s epoch using the continuous wavelet transform (CWT) as implemented in the MATLAB toolbox (R2019b, Mathworks, Natick, CA). To eliminate the effects of mains contamination, a narrow band‐stop filter at 50 Hz and all harmonics to 650 Hz was applied prior to performance of the CWT. In the present analysis, a Morlet wavelet was employed at a density of 24 voices per octave over 9 octaves. The CWTs were further transformed to scaleograms (time‐frequency images) from the absolute value of the CWT and rescaled to be in dB per voice re 1 μV^2^. Scaleograms were further split into eight frequency bands, and very high frequency (VHF: 160–320 Hz) and ultra‐high frequency (UHF: 320–640 Hz) were extracted for analysis. A summary spectrum over the whole epoch was also computed.

## 3 RESULTS

### 3.1 BrAC levels and neurological assessment

About 30 mins after the 1st dose of 20 g EtOH, after the 2nd block and just prior to the 2nd dose, the BrAC gave a BAC estimate of 0.05%. Neurological examination did not show any significant dysmetria nor gaze‐evoked nystagmus and speech appeared normal. 30 mins after the 2nd dose, and 20 mins after the start of the 3rd block, the BrAC gave a BAC estimate of 0.14%. A second neurological examination at this stage indicated mild dysmetria, fine gaze‐evoked nystagmus and mild dysarthria as well as a positive affective change. Following the 4th block, about 1 h and 20 mins after the 2nd dose, when the volunteer was temporarily disconnected from the recording apparatus, a heel/toe gait test showed noticeable unsteadiness, that is, ataxia. At this time the BrAC gave a BAC estimate of 0.09%. At the period of the 5th and last block, 2 ½ h after the 2nd dose, the BrAC gave a BAC estimate of 0.08%. By the time of discharge from supervision, 4 ½ hours after the 2nd dose, speech and heel/toe gait had returned to normal.

### 3.2 Acceleration, posturography, evoked potentials, EEG power, and EMG results

**FIGURE 2:**
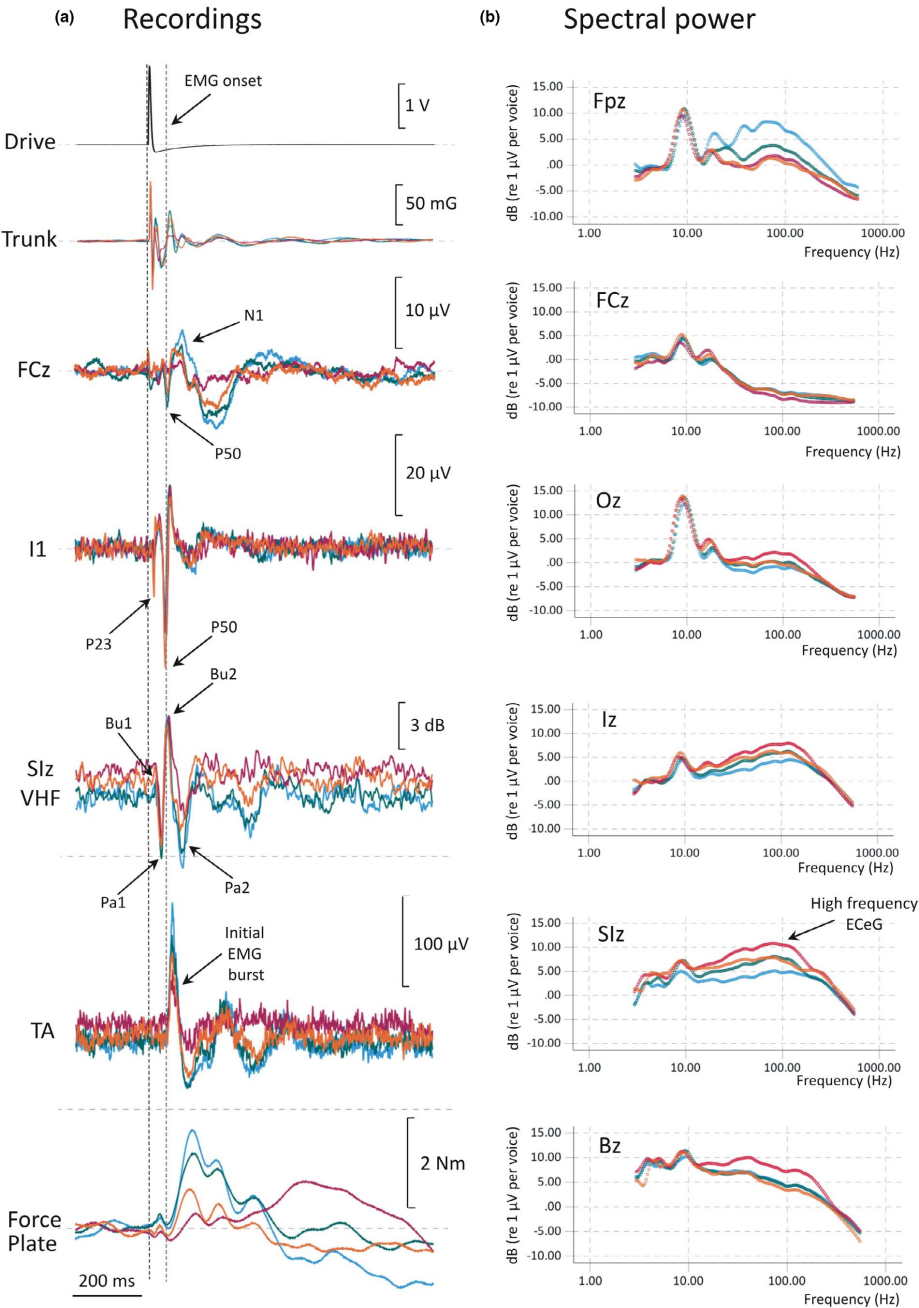
Effects of the mini perturbation: (blue) before EtOH, (green) after 55 mL 46% EtOH (or 20 g EtOH, two standard drinks), (red) after 200 mL 46% EtOH and (orange) after 2 ½ h recovery. (a) Recordings of trunk acceleration, EEG, ECeG, EMG and force plate 200 ms before and 800 ms after onset of stimulation. (b) Effects of EtOH on spectral power at six midline locations, Fpz, FCz, Oz, Iz, SIz and Bz. High frequency ECeG is maximal at SIz and increases with dose of EtOH. In contrast, high frequency power from frontalis muscle EMG at Fpz is reduced with the dose of EtOH.

Figure 2a shows the input drive, truncal acceleration, and induced force platform response along with cerebral/cerebellar responses and rectified EMG response from the lower limb muscles before EtOH (blue), after 1st dose (green), after 2nd dose (red) and 2 ½ h after 2nd dose (orange). Consistency of the backwards lean across conditions is confirmed by the baseline moment prior to stimulation. In the non‐intoxicated state the reflex to perturbation consists of a sharp excitation in TA activity with onset between 54 and 58 ms (marked in Figure 2a, Table 1) and peak between 70 to 73 ms after stimulus onset, followed by an inhibitory period with a trough between 106 and 112 ms (Table 1). Two subsequent distinct excitation/inhibition cycles occurred with peaks at about 220 and 390 ms. The TA excitation/inhibition results in a positive (i.e. forward) moment about the Y‐axis of the force plate, which acts to counter the backwards perturbation, peaking shortly after the TA trough at about 126 ms.

**TABLE 1:** Effect of EtOH on cerebral, cerebellar and postural responses, latencies (ms).

	Before	Dose 1	Dose 2	Recovery 1	Recovery 2	Mean (SD)
*Potentials*						
P23 Iz	21	22	21	23	21	21.6 (0.9)
N33 Iz	33	33	35	35	36	34.4 (1.3)
P50 Iz	51	52	55	52	52	52.4 (1.5)
P50 FCz	56	56	57	58	58	57 (1.0)
N100 FCz	98	97	93	100	95	96.6 (2.7)
*Power*						
Bu1	22	26	24	26	23	24.2 (1.8)
Pa1	39	40	40	41	38	39.6 (1.1)
Bu2	55	57	61	58	55	57.2 (2.5)
Pa2	100	97	106	105	95	100.6 (4.8)
*EMG*						
TA L onset	54.0	55.4	59.4	59.4	57.0	57.0 (2.4)
TA L peak	70.5	71.3	75.3	64.9	72.1	70.8 (3.8)
TA L trough	106.4	112.0	115.0	115.2	114.4	112.6 (3.7)
TA R onset	58.0	60.2	61.0	61.0	62.6	60.6 (1.7)
TA R peak	72.6	69.7	71.3	65.7	70.5	70.0 (2.6)
TA R trough	111.7	115.2	117.0	117.6	117.6	115.8 (2.5)
*Force*						
My peak	126	128	137	136	124	130.2 (5.9)

Electrodes over the cerebellum showed characteristic axial‐evoked, short latency evoked potentials (CEPs) maximal at the Iz and SIz levels comprising a double P‐N wave with latencies of 21/33 ms and 51/66 ms. The initial P23/N33 (Govender et al., 2024) occurs well before the onset of EMG while the later P50/N66 co‐occurs with the initial TA EMG burst (54–73 ms). The CEP was accompanied by characteristic induced changes in spontaneous activity, comprising an initial double burst/pause (Bu1/Pa1, Bu2/Pa2) in high‐frequency ECeG with peak latencies 22/39 ms and 55/100 ms, followed by a series of later bursting/pausing in the ECeG. As with the CEPs, the Bu1/Pa1 occurs prior to the initial TA EMG excitation while the Bu2/Pa2 overlaps with the 1st TA excitation/inhibition cycle, but with transition in Bu2/Pa2 occurring at about 70 ms, thus preceding the trough in TA by about 30 ms. At the cerebral level, an axial or postural EP was observed at the vertex (here FCz), with a “P50” at 56 ms, just after the cerebellar P50 (analogous to the P54 in Govender et al., [2024]) followed by long latency N1‐P2‐N2 waves at about 100, 170 and 300 ms.

With administration of EtOH we observed a severe disruption to the postural reflex, especially after the 2nd dose (total 200 mL 46% EtOH), with the initial TA burst amplitude about half that of the non‐intoxicated state, even with a small increase in background contraction, and the secondary cycles of TA excitation/inhibition abolished. These resulted in the virtual elimination of initial rapid forward transfer of weight, replaced by a much more sluggish transfer, peaking some 500 ms after perturbation. The cerebral EPs were also severely disrupted, with the cerebral P50 virtually abolished (Table 2). The recordings from over the cerebellum, however, showed contrasting changes following EtOH administration. The SL CEPs in the Iz/SIz region were virtually unchanged, if anything rebounding briskly during recovery, but the high frequency spontaneous and induced ECeG was severely disrupted, paralleling that of the leg EMG. There was a general increase in high frequency ECeG power with increasing dose in the Iz/SIz region (Figure 2b), and the later pause‐bursting became progressively more disrupted following Bu1/Pa1.

**TABLE 2:** Effect of EtOH on cerebral, cerebellar and postural response amplitudes.

	Before	Dose 1	Dose 2	Recovery 1	Recovery 2	Mean (SD)
*Potentials (μV)*						
P23 Iz	4.6	3.7	4.8	8.2	9.6	6.2 (2.6)
N33 Iz	6.1	5.8	8.2	6.8	6.7	6.7 (0.9)
P50 Iz	13.3	15.8	16.4	13.9	22.1	16.3 (3.5)
P50 FCz	3.6	5.1	0.2	3.1	5.1	3.4 (2.0)
N100 FCz	6.0	4.0	1.7	2.0	2.5	3.2 (1.8)
*UHF Power (dB)*						
Resting level	−1.7	−1.1	0.10	0.39	0.38	−0.39 (0.96)
Stimulus tonic level	−1.4	−2.1	−1.6	−0.72	−0.95	−1.4 (0.54)
Tonic reduction	+0.30	−1.0	−1.7	−1.1	−2.8	−1.3 (1.1)
Bu1	+1.4	+1.3	+1.3	+0.36	+1.2	+1.1 (0.43)
Pa1	−3.7	−4.0	−3.9	−5.1	−4.3	−4.2 (0.55)
Bu2	+5.5	+4.6	+4.2	+3.0	+4.6	+4.4 (0.91)
Pa2	−4.4	−2.2	−2.8	−2.4	−3.3	−3.0 (0.88)
*VHF Power (dB)*						
Resting level	4.5	5.8	7.6	7.7	7.6	6.6 (1.4)
Stimulus tonic level	3.4	3.3	5.2	4.9	4.3	4.2 (0.86)
Tonic reduction	−1.2	−2.5	−2.4	−2.8	−3.3	−2.4 (0.78)
Bu1	+0.74	+1.2	+0.57	+0.33	+1.2	+0.81 (0.39)
Pa1	−2.9	−4.8	−4.9	−6.2	−4.5	−4.7 (1.2)
Bu2	+5.9	+5.3	+4.3	+3.5	+4.2	+4.6 (0.95)
Pa2	−4.9	−3.9	−3.4	−2.7	−3.4	−3.7 (0.81)
*EMG (μV)*						
TA L bg	89.6	91.7	113.5	101.8	96.3	98.5 (9.6)
TA L peak	249.0	205.9	166.0	204.1	198.4	204.7 (29.6)
TA L trough	29.7	28.1	74.3	47.5	44.5	44.8 (18.6)
TA L pk (dB)	18.5	7.0	3.3	6.0	6.3	8.2 (5.9)
TA R bg	49.5	51.4	60.8	67.6	53.7	56.6 (7.5)
TA R peak	176.0	155.0	129.9	130.8	137.5	145.8 (19.6)
TA R trough	15.1	15.7	40.6	37.5	21.4	26.1 (12.1)
TA R pk (dB)	11.0	9.6	6.6	5.7	8.1	8.2 (2.2)
*Force (Nm)*						
My peak	2.15	1.64	0.33	0.64	0.86	1.1 (0.75)

After 2 ½ h following the 2nd dose, significant recovery was observed. The TA excitation/inhibition cycling was mostly restored and the associated rapid transfer of weight was recovered to about half that of the non‐intoxicated state. The cerebral P50 was restored, though there remained still some disruption to the axial N2. The high‐frequency ECeG power over the Iz/SIz level was still abnormally high (Figure 2b), especially at rest (Table 2), but the burst/pausing was partially restored, again paralleling that of the TA activity.

## 4 DISCUSSION

In the present case study, we recorded from over the cerebellum and cerebrum during a postural perturbation and associated reflexive response before and after administration of EtOH. EtOH disruption of reflexive and voluntary action has been very well described (Mitoma et al., 2021; Rossi, 2023), with some notable case studies highlighting in particular EtOH‐induced cerebellar physiological symptoms (Marsden et al., 1970; Traub et al., 1980). As noted above, the neurochemical mechanisms of EtOH‐induced cerebellar dysfunction have been widely articulated.

The particular postural reflex we employed here has also been well‐studied in healthy human participants (Colebatch et al., 2016; Govender et al., 2024; Todd et al., 2021). We have previously shown that stimulation of neck muscle afferents can also evoke this short latency postural response associated with a preceding cerebellar potential with a similar latency to that found here (P22) (Todd et al., 2022). Further, the P23 potential is correlated with the size of the following response in TA, although unlike the postural reflex itself, it is not altered by posture (Govender et al., 2022, 2024). The initial postural response component could thus be either transcerebellar or modulated by the P23 associated cerebellar activity. A delay of around 30 ms would be expected between cerebellar and leg EMG responses, allowing for central and peripheral delays (Jeyakumar et al., 2018).

The available evidence, including the latency, surface positivity and magnitude, supports the CEPs and associated pause‐bursting in high frequency ECeG as being CF responses and post‐CF pausing (Eccles et al., 1967; Todd et al., 2018b). The initial P23‐N33 and associated Bu1/Pa1 preceding the TA excitation/inhibition most likely is a CFR to axial stimulation via neck muscle spindle afferents with reticular targets for modulation of the reticular generated reflex from the same afferent input (Eccles et al., 1967; Todd et al., 2022). The second larger cerebellar P50/N66 and associated Bu2/Pa2 co‐occurring with the TA excitation/inhibition is likely an upstream manifestation of the reticular generated reflex via the reticulo‐olivary tracts (De Zeeuw et al., 1998; Eccles et al., 1967), and hence a secondary CFR, following the initial P23‐N33 CFR, which may play a role in sharpening the later components of the postural reflex. This is accompanied by the cerebral component of the ascending reticular activating system (ARAS) via the thalamus for which the cerebral P50 has been described (Skinner et al., 2004). Unlike the cerebral ARAS, its ascending cerebellar CF counterpart is more robust to EtOH disruption, but the cerebellar outputs, as manifest in ECeG, and likely underlying SS activity are not spared, hence the observed disruption here.

The changes in recorded activity we observed here in this case after administration of EtOH cannot be interpreted as anything other than being of central origin. Neck or scalp muscle EMG does not show these behaviors, and in the present data scalp EMG at Fpz was reduced with EtOH. It appears that CF afferent input to the cerebellum is preserved with mild degrees of intoxication while cerebellar output ‐ postural reflexes to axial stimulation ‐ is significantly attenuated. This indicates an action of alcohol potentially occurring at the level of the Purkinje projection to cerebellar nuclei.

We believe that these observations support the case for the viability and value of non‐invasive electrophysiology of the human cerebellum. Given that currently the physiology of the human cerebellum is still poorly understood, these insights should prove of both basic and clinical value.

## AUTHOR CONTRIBUTIONS

**Neil P. M. Todd:** Conceptualization; data curation; formal analysis; investigation; methodology; supervision; validation; visualization. **Sendhil Govender:** Investigation; visualization. **James G. Colebatch:** Conceptualization; data curation; investigation; methodology; project administration; resources; supervision; visualization.

## FUNDING INFORMATION

This study was not funded by any grant awarding body.

## CONFLICT OF INTEREST STATEMENT

We confirm that none of the authors has any conflicts of interest.

## ETHICS STATEMENT

This study received ethical approval from the South Eastern Sydney Local Health District Human Research Ethics Committee.

## Supporting information


Data S1.

## Data Availability

Data available on reasonable request.
