# Supplementary material for: Effects of ethanol on non‐invasively recorded cerebellar, cerebral, and postural responses to axial perturbation: A case study
Source: Physiol Rep. 2026 Apr 29;14(9):e70897. doi: 10.14814/phy2.70897 (PMC13128517; doi:10.14814/phy2.70897)
Supplement: Supplementary file 1 — Data S1. [file PHY2-14-e70897-s001.docx]

**CARE Checklist (Case Report Guidelines)**

**Manuscript Title:** Effects of ethanol on non-invasively recorded cerebellar, cerebral and postural responses to axial perturbation: A case study
**Authors**:**** Neil PM Todd, Sendhil Govender and James G Colebatch

| Item | Checklist Item | Manuscript Section & Location with Line Numbers |
| --- | --- | --- |
| **1** | **Title** | **Title Page, lines 1-2** (Includes "case study") |
| **2** | **Keywords** | **Keywords** (Ethanol, Cerebellum, ECeG, Postural Reflex) |
| **3a** | **Abstract: Background** | **Abstract**, lines 13–14, 18-22 |
| **3b** | **Abstract: Case Summary** | **Abstract**, lines 14–17, 22-26 |
| **3c** | **Abstract: Conclusion** | **Abstract**, lines 27–28 |
| **4** | **Introduction** | **Section 1**, entire section (Rationale/Validity of non-invasive ECeG) |
| **5a** | **Patient Demographics** | **Section 2.1**, line 78 (64-year-old male, 86 kg) |
| **5b** | **Primary Symptoms** | **Section 2.1**, lines 78-80, 86-97 (Healthy status; symptoms induced by EtOH) |
| **5c** | **Medical/Family History** | **Section 2.1**, lines 78-80 (No prior neurological/balance dysfunction) |
| **5d** | **Past Interventions** | **Section 2.1**, line 79 (Subject known from years of recording) |
| **6** | **Clinical Findings** | **Section 3.1,** lines 150-162 (Neurological exam results at BrAC 0.05% and 0.14%) |
| **7** | **Timeline** | **Section 2.1**, lines 86-89 (5 blocks over 3.5 hours detailed) |
| **8a** | **Diagnostic Methods** | **Sections 2.1–2.5, lines 94-148** (Detailed neurological examinations, perturbations, posturography, EEG/ECeG protocol) |
| **8b** | **Diagnostic Challenges** | **Section 1**, para 1, lines 32-47 (Discussion of non-invasive cerebellar EEG recording difficulties) |
| **8c** | **Diagnosis** | **Section 3.1, lines 150-162** (Acute ethanol intoxication confirmed by BrAC) |
| **8d** | **Prognosis** | **Section 3.1 & 3.2,** lines 160-162, 205-211 (Reversible nature of pharmacological effect) |
| **9a** | **Intervention (EtOH)** | **Section 2.1**, lines 86-89 (Oral ingestion protocol) |
| **9b** | **Administration (Dose)** | **Section 2.1**, lines 87-88 (Initial 20g dose, i.e. 55 ml of 46% EtOH; total 200 ml of 46% EtOH) |
| **9c** | **Intervention Changes** | **Section 2.1**, lines 82-89 (Sequence of 5 recording blocks) |
| **10a** | **Outcomes (Clinician)** | **Section 3.1, lines 150-162** (Nystagmus, dysarthria, and gait/ataxia assessment) |
| **10b** | **Outcomes (Patient)** | **Section 3.1, line 156** (Positive affective change noted) |
| **10c** | **Adverse Events** | **Section 3.1, lines 150-162** (Temporary ataxia; fully resolved at 4.5 hours) |
| **11a** | **Discussion: Strengths** | **Section 4**, para 4, lines 242-248 (Validity of non-invasive ECeG ) |
| **11b** | **Discussion: Literature** | **Section 4**, lines 215-239 (Citations 13 - 28) |
| **11c** | **Discussion: Conclusion** | **Section 4**, para 5, lines 249-252 (Case for viability of human cerebellar electrophysiology and clinical value) |
| **12** | **Patient Perspective** | **Section 2.1,** line 78 (Subject is an author; perspective is inherent in report) |
| **13** | **Informed Consent** | **Section 2.1**, lines 80-82 (Ethics approval and informed verbal consent) |
